# Expression of scavenger receptor A in rat’s liver tissue during acute obstructive cholangitis and its significance

**DOI:** 10.1186/s40064-016-2222-5

**Published:** 2016-05-12

**Authors:** Akanand Singh, Kang Yang, Zuojin Liu, Jianping Gong

**Affiliations:** Chongqing Key Laboratory of Hepatobiliary Surgery and Department of Hepatobiliary Surgery, The Second Affiliated Hospital, Chongqing Medical University, No. 76 Linjiang Road, Yuzhong District, Chongqing, 400010 People’s Republic of China

**Keywords:** Acute obstructive cholangitis, Scavenger receptor A, Endotoxin, Cytokine, Endotoxin hepatic injury

## Abstract

**Objective:**

Acute obstructive cholangitis (AOC) is a severe disease that leads to sepsis, shock and multiple organ dysfunction syndrome. Liver is the largest and the major organ for the defense mechanisms during the sepsis. Our aim was to investigate the expression of scavenger receptor A (SRA) in rat’s liver tissue during AOC and its relation with the inflammatory mediators and hepatic injury caused by endotoxins.

**Methods:**

Ninety Wistar rats were randomly divided into three equal groups. In group one, the choledochus were ligagted and *Escherichia coli* O_111_B4 injected into it to produce AOC model. Group two had choledochus ligated and group three had sham surgery. Six animals from each group were sacrificed at 0, 3, 6, 12 and 24 h for comparative analysis of endotoxins, tumor necrosis factor-alpha, interleukin-6 in plasma and expression of SRA protein and SRA mRNA in liver tissue. The P < 0.05 was considered significant. Ethical approval was obtained.

**Results:**

The plasma endotoxins, tumor necrosis factor-alpha and interleukin-6 levels in AOC rats increased progressively with time. The SRA protein and SRA mRNA expression decreased with time. The differences among groups were significant (P < 0.01). Liver histopathology showed gradually aggravated hepatic injury with neutrophil infiltration, degeneration and necrosis of hepatocytes.

**Conclusion:**

In AOC, the expression of SRA in liver gradually decreased with progressive increase in level of plasma endotoxins and hepatic injury suggesting its importance in the defense mechanism.

## Background

Liver is vital organ which has many functions including immunological tolerance for foreign antigens. Liver diseases are major health problems with significant mortality (Guan and He 2013). Acute obstructive cholangitis (AOC) commonly leads to sepsis, septic shock and multiple organ dysfunction syndrome (MODS) (Takada et al. 2013). Acute obstructive cholangitis (AOC) is a type of cholangitis with the symptom of right upper abdominal pain, jaundice, chills with fever. In severe cases of AOC, sign of central nervous system such as lethargy, disorientation, or coma, combined with septic shock are involved (Liao et al. 2009; Gong et al. 2002). It easily lead to systematic inflammatory response syndrome (SIRS) and MODS. Mortality rate of AOC is high if delayed in treatment which make the high mortality in nonmalignant diseases of biliary tract. Intervention for drainage of obstructed biliary tract is the therapeutic principle. However, there is still a high mortality even after the drainage as it already leads to sepsis and MODS in early stage of this disease. So, postoperative management is still very vital.

Liver is the largest organ for defense in sepsis (Hilliard et al. 2015). Kupffer cells play vital role in defense by clearing bacterium and endotoxin (Bilzer et al. 2006; Hutchins et al. 2013). They also have important role in the pathogenesis of hepatic injury in sepsis and MODS (Sato et al. 2014; Kim et al. 2011; Rivera et al. 2007). Although pathological and immunological mechanism of hepatic injury due to inflammatory diseases have not been completely understood, activation of hepatic endotoxin is critical event in inflammatory process (Heymann et al. 2015). Scavenger receptor A (SRA) is a transmembrane glycoprotein and mainly distributed in hepatic Kupffer cells (Prabhudas et al. 2014). The SRA reflects defense reaction that mediates macrophage clearance and inactivation of endotoxin (Kelley et al. 2014).

We aim establish animal model of AOC in Wistar rats to investigate the expression of SRA and its relations to endotoxin, tumor necrosis factor (TNF-α), interleukin (IL-6) and hepatic injury. The histological changes in Kupffer cells will provide evidence for its changing role from immune defense to inflammatory response cells during AOC. These observations may provide basis of therapeutic regimen targeted to protect liver cell damage during AOC, sepsis and MODS.

## Materials and methods

### Animal

All experimental protocols described in this study were approved by the research ethics review committee of the Second Affiliated Hospital of Chongqing Medical University and comply with the Chinese government guidelines including use of animals. Ninety Wistar rats of both sexes weighing 200 ± 20 g were divided randomly into three groups of 30 rats. First group was the acute obstructive cholangitis group (AOC) whose choledochus were ligated and 0.2 ml of *Escherichia coli* O_111_B4 (5 × 10^9^ CFU/ml) injected into it to reproduce animal model of AOC. The second was bile duct ligation group (BDL) whose choledochous were ligated and 0.2 ml normal saline injected into it and third was the sham operation group (SO). Randomly six rats from each group were sacrificed at 0 (immediately), 3, 6, 12 and 24 h respectively. Plasma and liver tissues were extracted and stored at −20 and −70 °C for later use.

### Methods

#### Endotoxin concentration in plasma

The Limulus Amebocyte Lysate assay kits manufactured by Shanghai Yihua Medical Technology Inc China were used to detect the concentrations of endotoxin in plasma. The presence of the endotoxin was detected at 545 nm using the Bio-tek Kcjunior microplate reader.

#### TNF-α and IL-6 levels in plasma

The levels of TNF-α and IL-6 in plasma were measured with enzyme-linked immunosorbent assay (ELISA) kits from Boster Inc, Wuhan, China to determine rat cytokines following manufacturer’s instructions. The cytokines were detected at 450 nm still using the Bio-tek Kcjunior microplate reader.

#### Expression of SRA protein

The expression of SRA protein in liver tissue was assayed by standard immunohistochemistry examination. The first antibody was goat anti-mouse SRA monoclonal antibody, which was bought from Santa Cruz Inc. The second antibody was rabbit anti-goat antibody from Wuhan Boster Inc. The liver tissues were observed under light microscope at 400 magnification to calculate average Immunohistochemistry (IHC) (+) Masculine Cell Population (MCP) by randomly selecting five visual fields to count IHC (+) MCP.

#### Expression of SRA mRNA

The expression of SRA mRNA in liver tissue was measured by real time polymerase chain reaction (RT-PCR). The primer of SRA mRNA and the primer of β-actin mRNA as control were designed according to method of Singh A et al. (Singh et al. 2015). The images of RT-PCR amplification products from gel electrophoresis were analyzed in automatic BioRad Image Pro-Plus system for expression of SRA mRNA. The relative expression of SRA mRNA was equal to IOD_SRA_/IOD_β-actin_.

#### Histopathological study in liver tissue

The liver tissue samples were fixed in 10 % neutral-buffered formalin. After 48 h of fixation, liver tissues were embedded in paraffin. 4 µm sections were sectioned, and stained with hematoxylin and eosin for conventional histopathological evaluation.

### Statistical analysis

Results are expressed as mean ± SEM. Statistical analysis were performed using software of SPSS 20.0. The difference among each group were analyzed by t test. A *P* value below 0.05 was considered to be significant.

## Results

### The changes of plasma endotoxin levels

**Fig. 1:**
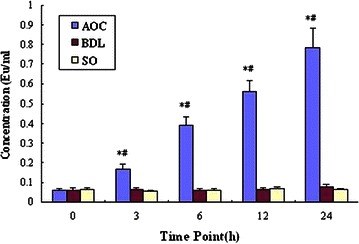
The comparison of plasma endotoxin concentration in various time points. Compared with Bile Duct Ligation (BDL) and Sham Operation (SO) groups, *P < 0.01; Compared with same group, ^#^P < 0.01

The plasma endotoxin concentrations in AOC group were higher at 3 h after surgery and increased progressively reaching peak at 24 h. The levels of endotoxin in BDL group increased slightly at 24 h, and no increase was seen in SO group (Fig. 1). The increase in level endotoxin in AOC group was significant compared to BDL and SO group (*P* < 0.01).

### The changes of plasma TNF-α and IL-6 levels

**Fig. 2:**
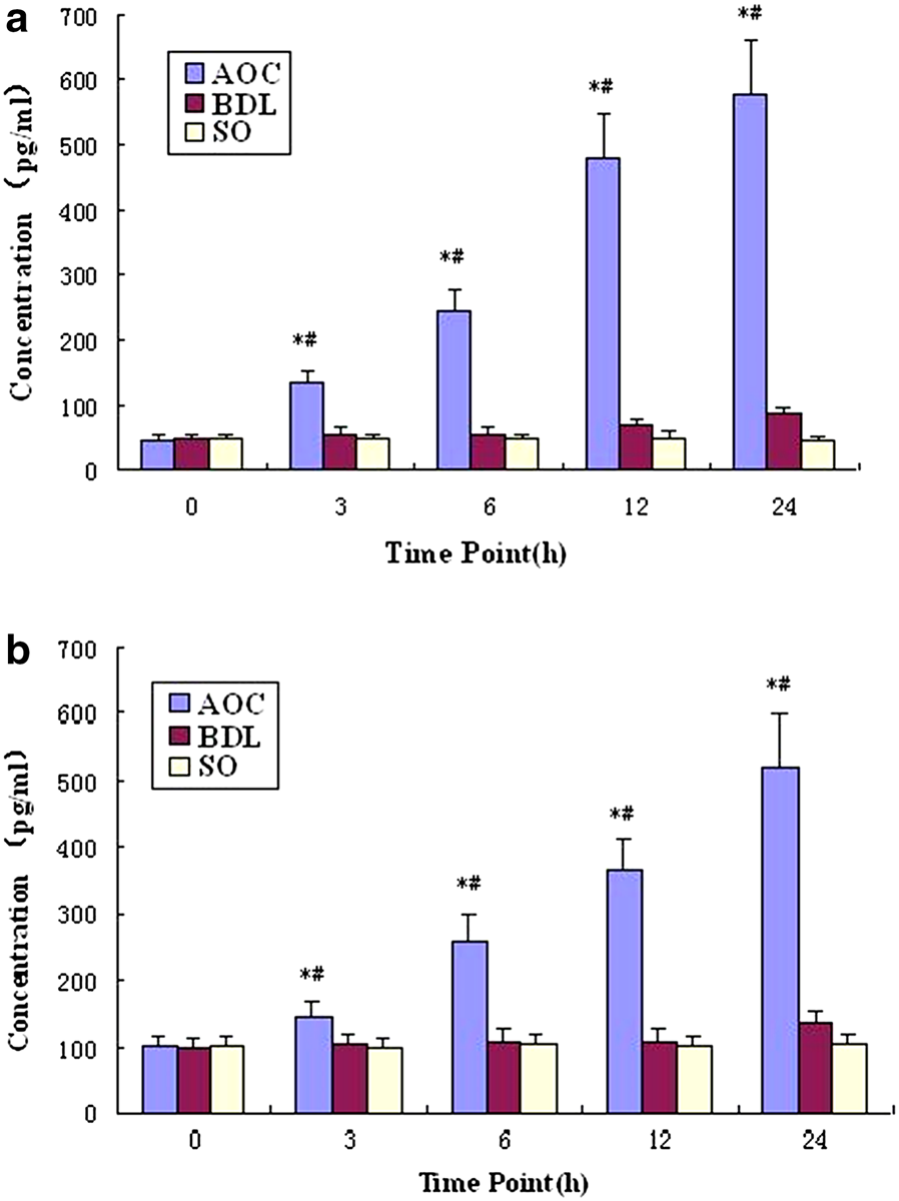
The comparison of **a** plasma TNF-α and **b** plasma IL-6 concentrations in various time points. Compared with Bile Duct Ligation (BDL) and Sham Operation (SO) groups, *P < 0.01; compared with same group, ^#^P < 0.01

The plasma TNF-α in AOC group had early increase at 3 h, IL-6 started at 6 h and continued to rise reaching peak at 24 h. There was mild increase in BDL and no rise in SO group (Fig. 2a, b). The rise in AOC group was significant (*P*<0.01) compared to BDL and SO groups.

### The expression of SRA protein in liver tissue

**Fig. 3:**
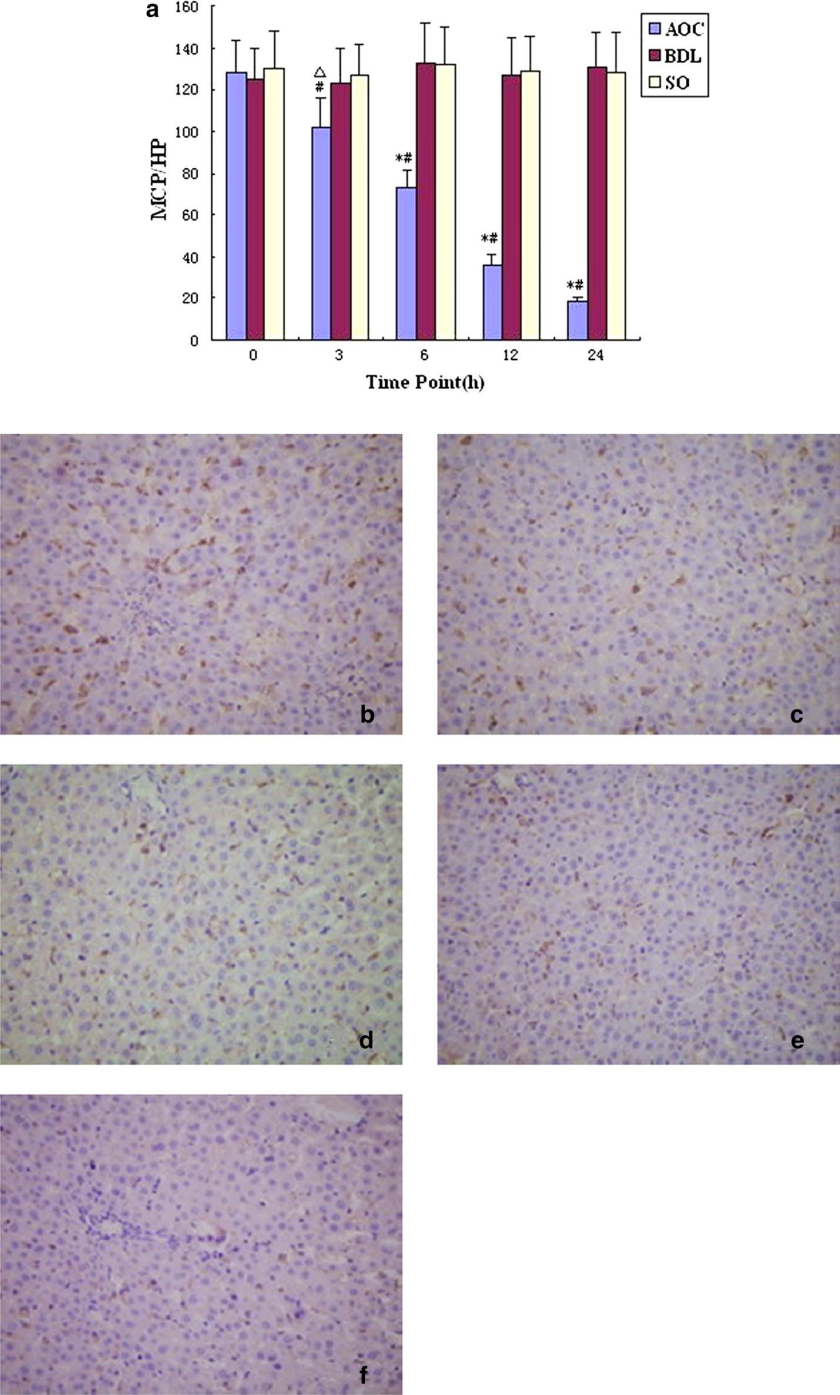
**a** The comparison of scavenger receptor A (SRA) expression in various time points (IHC) mcp/HP. Compared among acute obstructive cholangitis (AOC) group, Bile Duct Ligation (BDL) group and Sham Operation (SO) groups, *P < 0.01, ^△^P < 0.05; compared with same group, ^#^P < 0.01. **b** Kupffer cells’ SRA expression in acute obstructive cholangitis (AOC) groups at 0 h (SP × 400). The *brown* positive products of SRA expression are mainly located on the surface of Kupffer cells and diffusely distributed in liver tissue. **c**–**f** Kupffer Cells’ SRA expression in liver tissue in acute obstructive cholangitis (AOC) group at 3, 6, 12, 24 h (SP × 400). SRA expression products reduce with prolonged experimental time

The expression of SRA protein in liver tissue of AOC group was higher compared with BDL and SO groups (*P*<0.01) (Fig. 3a). Immunohistochemistry showed that the brown positive products of SRA expression were mainly located on the surface of Kupffer cells and were diffusely distributed inside liver tissue in AOC group at 0 h (Fig. 3b). The expression of SRA in AOC group had decreased at 3 h and gradually descended with prolonged experimental time. It was very obvious at 24 h (Fig. 3c–f).

### The expression of SRA mRNA in liver tissue

**Fig. 4:**
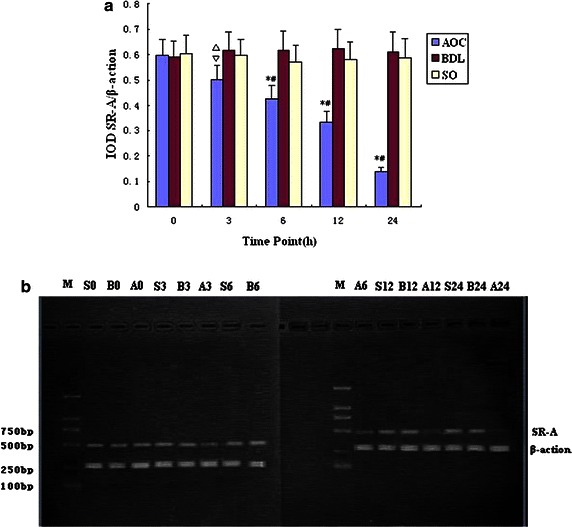
**a** Relative expression of scavenger receptor A (SRA) mRNA in liver tissue in various time points (IODSRA/IODβ-action). Compared among acute obstructive cholangitis (AOC) group, Bile Duct Ligation (BDL) group and Sham Operation (SO) group, *P < 0.01, ^△^P < 0.05; compared with same group, ^#^P < 0.01, ^▽^P < 0.05. **b** Relative expression of SRA mRNA in various time points by RT-PCR. SRA mRNA expression in acute obstructive cholangitis (AOC) group progressively decrease with prolonged experimental time. *M* marker; *A*, *B*, *S* AOC, BDL, Sham Operation (SO) group, subsequent number stand for time point

RT-PCR showed that the expression of SRA mRNA in AOC group had descended at 3 h and decreased progressively with prolonged experimental time. At 24 h, compared with the BDL group and SO group, AOC group have very significant differences. The changes in BDL and SO groups were not evident (*P* < 0.01). The expression of SRA in gene and protein gradually decreased with progressive increasing plasma endotoxin levels (Fig. 4a, b).

### Histopathological study in liver tissue

**Fig. 5:**
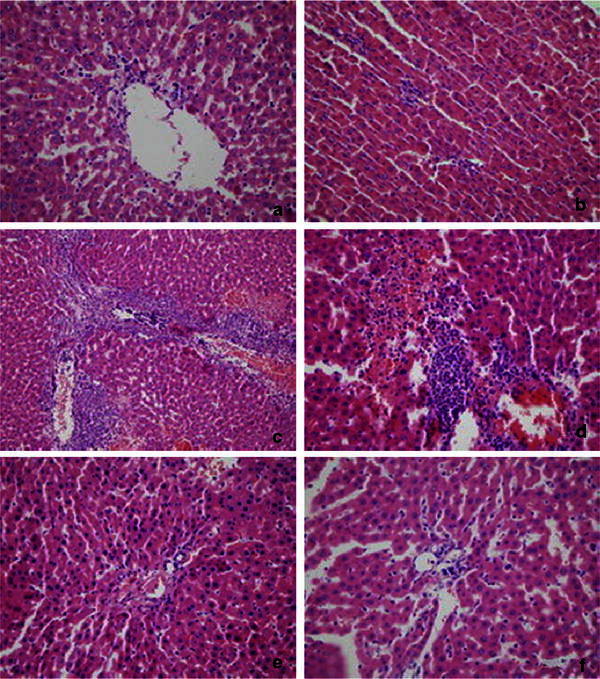
**a**–**d** Liver tissue in acute obstructive cholangitis (AOC) group at 3,6,12 and 24 h (HE × 400). Many inflammatory infiltration mainly consisting of neutrophil and expansion of central veins are found at 3 h. Focal inflammation are found in liver parenchyma at 6 h. Obvious expansion of portal area, massive mixing inflammatory cells infiltration are found at 12 h. Large-area hepatocyte degeneration and necrosis are found at 24 h. **e** Liver tissue in Bile Duct Ligation (BDL) group (HE × 400), Manipulus Inflammatory cells infiltration are found in portal area. **f** Liver tissue in Sham Operation (SO) group (HE × 400), no obvious histopathological changes are found in liver tissue

Histopathological features observed under light microscope showed gradual aggravation of hepatic injury in AOC group with infiltration of neutrophil in veins at 3 h (Fig. 5a), focal liver parenchyma at 6 h (Fig. 5b), portal area at 12 h (Fig. 5c) and large-area hepatocyte degeneration and necrosis at 24 h (Fig. 5d). In BDL group, few inflammatory cells were found in portal area and not in liver parenchyma (Fig. 5e). Histopathological changes were not seen in SO group (Fig. 5f).

## Discussion

After establishment of AOC rat model, we assayed plasma endotoxin level at various time points. We found the concentration of plasma endotoxin increased with prolonged time, which indicates the success of establishment of AOC animal models.

Immunohistochemistry staining showed the expression of SRA gradually decreases with experimental time which means, it gradually decreases with increasing lipopolysaccharide (LPS). In the meantime, the expression of SRA mRNA also progressively decreases with the increase of LPS. Furthermore, we found that SRA expression in mRNA was in accordance with that in protein. The expression of SRA in mRNA and protein gradually decreased with progressive increase in the level of plasma endotoxin. The expression of SRA mononuclear macrophages during endotoxemia has been widely reported (Ozment et al. 2012; Zhao et al. 2015). One recent study suggest that SRA is needed for LPS induced inflammatory responses in macrophages (Yu et al. 2012). SRA is considered multifunctional and has contribution in defense mechanisms (Zuo et al. 2013).

Sepsis or MODS remains one of the major causes of death in AOC (Huggett et al. 2014). Binding of LPS to macrophages could induce the release of cytokine and inflammatory mediators, leading to organ damage (Singh et al. 2015). In our study, the plasma level of cytokines, TNF-alpha and IL-6 in AOC group significantly increased at 6 h after operation and progressively increased with increasing level of plasma endotoxin. These findings indicate that inflammatory response is progressively enhanced in the liver tissue in AOC, which had positive relation with plasma endotoxin.

Kupffer cells are resident macrophages which reside within the lumen of liver sinusoids. They constitute 80–90 % of the tissue macrophages present in the body (Bilzer et al. 2006). The surface of Kupffer cell has different LPS receptors such as CD14, TLRs, CD11a/CD1lb/CD18, CD1lc/CD18, SRA, etc. These LPS receptors clear the bacterium and endotoxin of the blood receiving from portal vein. On the other hand, they also serve as mediators of inflammation, especially on endotoxin hepatic injury via releasing various cytokines (IL-6, TNF-α, etc.) (Sato et al. 2014). At present it is reported that Kupffer cells not only play important role in occurrence and development of liver diseases but also in liver’s ischemia–reperfusion injury (Suyavaran et al. 2015).

SRA, which is first described by Brown and Goldstein in 1970s is a superfamily of membrane-bond receptors that were initially thought to bind and internalized modified low-density lipoprotein (LDL), though it is currently known to bind to variety of ligands including endogenous proteins and pathogens (Goldstein et al. 1979). Currently, SRA is classified into 10 eukaryote families, defined as Classes A–J (Zani et al. 2015). SRA is mainly distributed in various kinds of tissue macrophages, particularly in hepatic Kupffer cells, spleen and lymph node macrophages. It is an important defensive receptor in macrophage surface, which can combine with bacterial LPS. It plays important roles in defensive reaction which mediates macrophages clearing and inactivating endotoxin.

In this study, correlation analysis showed that changes in the levels of TNF-alpha and IL-6 in plasma were negatively correlated with the expression of SRA mRNA and protein. Meanwhile, in one previous study, blocking expression of SRA on Kupffer cells could promote cytokine production. It is already reported that TNF-alpha and IL-6 has been related with endotoxin mediating inflammation and liver injury (Zhang et al. 2015; Zhou et al. 2015). The reduced SRA expression leads to decrease on clearing and inactivating endotoxin, while endotoxin activates more Kupffer cells (Xie et al. 2001; Jiang et al. 2003). Therefore, with participation of CD14 (another important receptor in surface of Kupffer cells, which is concerned with activation of Kupffer cells), Kupffer cells turn into effector cells. The release of TNF-α and IL-6 are increased, which result in more down-regulation of SRA expression and up-regulation of CD14 expression.

The LPS-induced hepatic injury gradually aggravated with prolonged experimental time in AOC group in our study. On Light microscope, the liver tissue showed different injuries characterized by infiltration of inflammatory cells, hepatocyte denaturation and necrosis. The degree of hepatic injury were related to endotoxin and correlated negatively with SRA. Therefore, in addition to surgical drainage of obstructed biliary tract, the control of Kupffer cell functions, especially the expression of SRA may have benefit in the management of AOC. This is an area of further research.

## Conclusion

In rat AOC model the expression of SRA from liver Kupffer cells decreased gradually with increasing plasma endotoxin, TNF-α and IL-6 leading to progressive liver injury of hepatocytes, suggesting the importance of SRA in the defense mechanism during AOC.
